# Evaluation of In Vitro Inhibitory Activity of Extracts of Garlic, Ginger, and Onion Against *Escherichia coli* and *Staphylococcus aureus* Isolated from Milk of Dairy Cows

**DOI:** 10.3390/vetsci12100947

**Published:** 2025-09-30

**Authors:** Hoang Thi Anh Phuong, Cara Robison, Pamela Lynn Ruegg

**Affiliations:** 1 Department of Veterinary Medicine, Tay Nguyen University, Dak Lak 63000, Vietnam; htaphuong@ttn.edu.vn; 2 Department of Large Animal Clinical Sciences, Michigan State University, East Lansing, MI 48824, USA; oconn107@msu.edu

**Keywords:** garlic, ginger, onion, bovine mastitis, antibiotic resistance

## Abstract

**Simple Summary:**

Bovine mastitis is a common disease in dairy cows that is often treated with antibiotics, but there is increasing concern that use of antibiotics on farms may contribute to the development and spread of resistant bacteria. Identification of non-traditional treatments may help to reduce potential development of antimicrobial resistance. The purpose of this study was to determine if extracts of garlic, ginger, and onion were able to inhibit growth of *E. coli* and *S. aureus* that had been isolated from milk samples of cows with mastitis. Fresh extracts of garlic, ginger, and onion were tested in vitro across a range of concentrations. Garlic inhibited growth of both organisms, but extracts of fresh ginger and onion were not able to inhibit growth. Future studies are needed to determine if garlic can reach an effective concentration in the mammary gland and efficacy in naturally occurring infections.

**Abstract:**

The purpose of this study was to identify the potential inhibitory effects of extracts of garlic, ginger, and onion on *Escherichia coli* and *Staphylococcus aureus*, which had been previously isolated from milk of dairy cows with mastitis. Garlic, ginger, and onions were crudely pressed, and the extracts were filtered and tested for their ability to inhibit bacterial growth at a wide range of concentrations, from undiluted to 1:512 (2^−9^). Their inhibitory properties were compared to positive controls containing ampicillin and ceftiofur, and negative controls containing only the nutrient medium and bacteria. Each plate contained quality control organisms *E. coli* ATCC 25922 and *S. aureus* ATCC 25923. The colorimetric microdilution method with resazurin as an indicator of bacterial growth was used to determine the minimum inhibitory concentrations. In addition, the minimum bactericidal concentrations of the extracts were assessed. The minimum inhibitory concentrations of garlic extracts were 1.56 µL/mL and 3.12 µL/mL for *E. coli* and *S. aureus*, respectively. The minimum bactericidal concentrations of garlic extract against *E. coli* and *S. aureus* were 12.5 and 25 µL/mL, respectively. For both ginger and onion, no inhibition was detected at the full concentration of the extracts, but garlic extract demonstrated in vitro inhibition against both *E. coli* and *S. aureus*. Future studies should evaluate the ability of garlic extracts to achieve an inhibitory concentration in milk and explore its potential activity in naturally infected mammary glands.

## 1. Introduction

Throughout the world, mastitis is the most common bacterial disease of lactating dairy cows and is almost always the most common reason that antibiotics are given to mature cows [1,2,3]. In developed dairy regions, enhanced surveillance of raw milk has resulted in considerable progress in reducing antibiotic residues in milk, but consumers and public health officials remain concerned about use of antimicrobials in dairy cattle. A survey of 1000 consumers in the United States reported that 70% of responders considered antibiotic usage in dairy cows to be a moderate-to-high threat to their own health [4]. Most of the concerns about antibiotic usage on dairy farms are based on the risk of emergence and spread of bacteria that are resistant to important antibiotics. Antibiotics are a limited resource and most of the antibiotics that are used in dairy cows are in the same classes that are used for treatment of people. The World Health Organization has classified antimicrobials based on their importance for treating bacterial diseases in humans [5], and “the highest priority, critically important antimicrobials” (CIAs) refer to antibiotics that are needed for treatment of serious bacterial infections affecting humans. Restrictions in usage of CIAs in veterinary medicine have been enacted by public health officials in many countries [6]. The continued pressure to conserve the existing antimicrobial classes has resulted in a strong emphasis on reducing antibiotic usage in food-producing animals, and an urgent need to identify alternative solutions to traditional antibiotics [7]. Botanical extracts with potential antimicrobial activity are increasingly being considered as alternatives that may reduce risks of development of antibiotic resistance [8]. Many plant-based antimicrobials have been assessed, and some may have potential roles as livestock therapeutics based on their antibacterial, antioxidant, or immunological properties [9,10,11].

Garlic (*Allium sativum*), ginger (*Zingiber officinale*), and onion (*Allium cepa*) are widely used plants known to possess antimicrobial properties. Garlic, a member of the genus *Allium* in the family *Alliaceae*, is among the most used spices worldwide [12]. In some regions, it is incorporated into livestock diets to enhance growth and improve resistance to disease [13,14]. The antimicrobial activity of garlic is primarily attributed to its bioactive compounds, such as allicin, diallyl disulfide, ajoene, flavonoids, polysaccharides, and saponins [15]. Ginger, belonging to the family *Zingiberaceae*, is both a culinary spice and a traditional medicinal plant [16]. Its antibacterial, antioxidant, antifungal, and anti-inflammatory effects are linked to its active constituents, including zingiberene, flavonoids, phenolics, gingerol, shogaol, and zingerone [17,18,19]. Onion, a member of the genus *Allium* in the family *Amaryllidaceae*, is also a staple food that is consumed throughout the world [6]. In livestock, onions have been reported to exhibit antibacterial, antifungal, and immune-enhancing effects [20,21], largely due to components such as quercetin, fructans, saponins, vitamin C, thiosulfinates, and cepaenes [22].

The initial step in evaluating botanical compounds as alternative antimicrobials is to assess their in vitro activity against common pathogens. The typical approaches for testing antibacterial properties of plant extracts include agar disc diffusion and broth or microdilution assays, with dilution methods generally considered to be more reproducible [23]. These methods determine the bacterial inhibition by measuring the growth turbidity, often using a colorimetric indicator. The resulting minimum inhibitory concentrations (MICs) provide a quantitative measure of the antimicrobial potency of the extracts against selected microorganisms. Once in vitro activity has been established, further research is required to evaluate whether inhibitory concentrations can be achieved at infection sites in live animals, as well as to confirm animal safety and assess potential food residue risks. The purpose of this study was to examine the in vitro antibacterial abilities of extracts of garlic, ginger, and onion against *E. coli* and *S. aureus*. We hypothesized that these extracts may be effective at inhibiting growth of *E. coli* and *S. aureus* that had been recovered from milk of bovine mastitis cases, and that their ability to inhibit bacterial growth would vary among the extracts.

## 2. Materials and Methods [23]

### 2.1. Location

This study was conducted from August to December 2024 at the College of Veterinary Medicine, Michigan State University, East Lansing, United States.

### 2.2. Extract Preparation

Fresh garlic, purple onions, and ginger were purchased from a commercial grocery store in Lansing, Michigan, and were peeled, rinsed with distilled water, drained, and then finely ground using a stone mortar and pestle. The resulting paste was transferred into sterile plastic bags, and the crude extract was pressed and collected into 15 mL sterile centrifuge tubes with secure caps and centrifuged at 3000 rpm for 5 min. The sedimented extract at the bottom of the tubes was transferred into sterile plastic containers through a sterile nylon filter cap with a pore size of 40 µm. Finally, the extract was drawn using a sterile syringe and passed through a 0.22 µm sterile syringe filter. The sterile extracts were prepared and tested for inhibitory properties within hours of extraction on the same day of preparation. After preparation, the extracts were inoculated on blood agar and incubated at 37 °C for 24 h under aerobic conditions to confirm sterility. No bacterial growth was observed for any extract.

### 2.3. Minimum Inhibitory Concentration (MIC)

The *E. coli* and *S. aureus* used in this study were previously isolated from milk samples of dairy cows affected with mastitis and had been stored at −80 °C. The isolates were a convenience sample from the cryopreserved collection containing >4700 isolates of the senior author (P.L.R.), and originated from quarter milk samples collected from cows with clinical or subclinical mastitis. The isolates were clinical isolates that had not been previously characterized for their resistance profiles nor for their virulence factors. The identity of all the isolates was originally confirmed using conventional microbiological methods and/or matrix-assisted laser desorption/ionization–time-of-flight mass spectrometry (MALDI-TOF-MS) [24,25]. To ensure purity of the isolates, before use the cryopreserved bacteria were inoculated onto blood agar and incubated at 37 °C for 24 h and then picked and regrown on blood agar.

Bacterial suspensions were performed following the CLSI guidelines [23]. Briefly, 3 to 5 colonies from plates with pure growth of each isolate were inoculated into tubes containing 5 mL of Luria–Bertani broth (LB, Miller, Appleton, WI, USA), vortexed, and incubated in a shaking incubator at 35 °C ± 2 for 3 to 4 h. The bacterial suspensions’ turbidity were adjusted to 0.5 McFarland using sterile 0.9% saline. Three replicates of each *S. aureus* (n = 10) and each *E. coli* (n = 10) were tested against each extract (onion, garlic, and ginger) for a total of 180 MIC tests.

Microdilutions were prepared, with each plate containing 12 columns and 8 rows (Table 1). Columns 1 to 10 were used to test the botanical extracts using twofold serial dilutions, starting with 50 µL of undiluted fresh crude extract. Columns 11 and 12 contained ampicillin (96–102% stated purity; Sigma-Aldrich, St. Louis, MO, USA) and Ceftiofur (97.6%; Sigma-Aldrich, St. Louis, MO, USA), which served as the positive controls. The antibiotic powders were reconstituted in sterile autoclaved water, with final concentrations of ampicillin at 2 µg/mL and ceftiofur at 1 µg/mL for the *S. aureus* tests, and ampicillin at 8 µg/mL with ceftiofur at 1 µg/mL for the *E. coli* tests, as defined by the CLSI guidelines [23]. The wells serving as negative controls included only the bacterial suspension, resazurin, and LB broth. Each isolate was replicated 3 times in consecutive rows of the plate. One row of each plate was reserved for testing the extracts against the quality control isolates *E. coli* ATCC 25922 and *S. aureus* ATCC 25923 (Supplementary Table S1).

A total of 50 µL of the appropriately diluted botanical extract was dispensed into the wells, followed by 30 µL of Iso-Sensitest broth (Oxoid, UK). Then, 10 µL of bacterial suspension was added and gently mixed. After 15 min, 10 µL of the 40% resazurin solution was added to each well [26].

**Figure 1:**
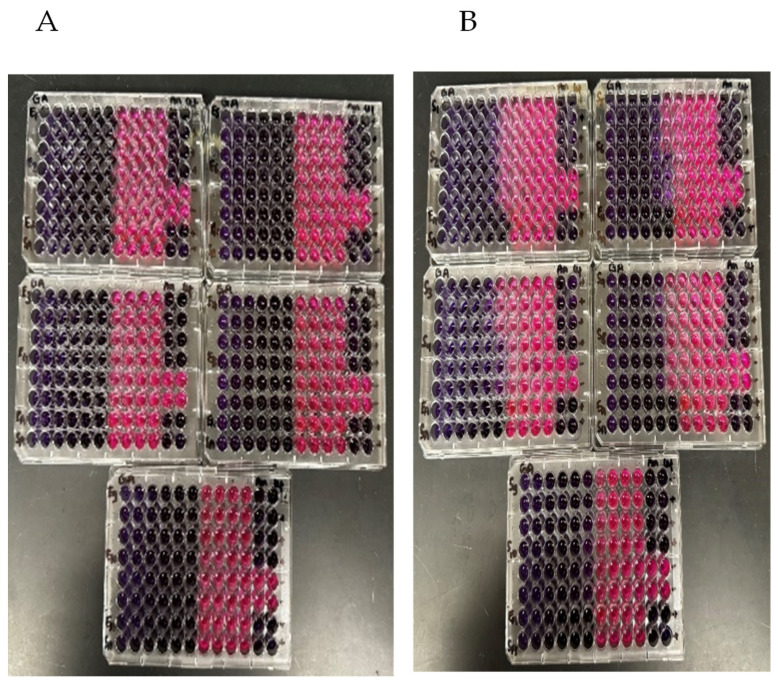
Example of results from the 96-well plates containing extracts of garlic after inoculation and incubation. Column (**A**) shows garlic extract tested against replicates of *E. coli*; (**B**) shows garlic extract tested against *S. aureus*. All the tests were performed using 3 replicates of 10 separate isolates of each organism. Each row contained sequential dilutions of the specified extract, the indicator resazurin, and the specified test organism, and the quality control organism or negative control (see Table 1 for layout of the plates). A blue/purple color indicates no bacterial growth within the well while pink (or colorless) indicates growth.

After incubation for 24 h, one researcher visually assessed the color changes in each well, and a change in color from purple to pink or colorless was considered indicative of bacterial growth (Figure 1). The color of the liquid in the wells was purple if bacterial growth did not occur, but turned pink or colorless when bacterial growth occurred. The lowest concentration at which the well remained purple was recorded as the MIC.

### 2.4. Minimum Bactericidal Concentration (MBC)

The minimum bactericidal concentration (MBC) was determined using a serial dilution method [27]. Each bacterial isolate was suspended in LB broth and adjusted to 0.5 McFarland. The suspensions were incubated at 37 °C for 3 to 4 h. Sterile test tubes (n = 10) were prepared, each containing 950 µL of LB broth. Then, 10 µL of the bacterial culture was added to tubes 1 through 9. Subsequently, 50 µL of each extract was serially diluted using twofold dilutions. The tenth tube, which served as the growth control, contained only LB broth. All the tubes were incubated at 37 °C for 24 h in a shaking incubator. Then, 100 µL from each tube was plated onto sterile tryptic soy agar (TSA; BD, Franklin Lakes, NJ, USA) and further incubated at 37 °C for another 24 h. The MBC was defined as the lowest concentration of the extract at which no visible bacterial growth was observed.

### 2.5. Statistical Analysis

Descriptive statistics were generated using R software (version R 4.4.1). The proportion of isolates inhibited at each concentration was calculated to describe the antibacterial activity in relation to the dilution levels.

A Kruskal–Wallis rank-sum test was conducted to evaluate the differences in the MBC values (log_2_-transformed) between *E. coli* and *S. aureus*. Heatmaps were employed to visualize the results, facilitating the interpretation of antibacterial trends and the ability of garlic extracts to inhibit growth among the different bacterial isolates (Supplementary Figure S1). Violin plots were combined with boxplots to illustrate the distribution of MICs and MBCs at log_2_ scale (Supplementary Figure S2).

## 3. Results

### 3.1. MIC of Extracts Against E. coli and S. aureus Isolates

The positive control wells containing ampicillin and ceftiofur inhibited all bacterial growth of the test isolates and the ATCC isolates, while bacterial growth occurred in all the negative control wells. Extracts of ginger and onion did not inhibit growth of either *E. coli* or *S. aureus* at any concentration tested. The garlic extracts inhibited growth of both *E. coli* and *S. aureus* (Table 1, Figure 1A,B). The MIC of garlic extract for all *E. coli* isolates was 1.56 µL/mL (5.0 log_2_). The MIC of garlic extract for the *S. aureus* isolates varied by one dilution and ranged between 1.56 (n = 4 isolates) and 3.12 (n = 6 isolates) µL/mL. Overall, the estimated MIC for both *E. coli* and *S. aureus* fell within the range of 1.56 to 3.12 µL/mL, depending on the bacterial isolate (Figure 1, Table 1). The Kruskal–Wallis test indicated that the MIC values varied between the two bacterial groups (χ^2^ = 8.14, df = 1, *p* = 0.004) (Supplementary Figures S1A and S2A).

### 3.2. MBC of Extracts Against E. coli and S. aureus Isolates

Like the MIC results, the log_2_(MBC) values indicated that *E. coli* was inhibited at one dilution less than *S. aureus*, with MBCs of 12.5 µL/mL and 25 µL/mL for *E. coli* and *S. aureus*, respectively (Supplementary Figures S1B and S2B). The bactericidal activity of garlic extract varied between *E. coli* and *S. aureus* (χ^2^ = 19, df = 1, *p* < 0.001). The ratios of the MBCs to MICs exceeded eight for both organisms, suggesting that garlic possesses bactericidal activity against both *E. coli* and *S. aureus*.

## 4. Discussion

Although many intramammary infections result in a spontaneous cure [28], bovine mastitis is frequently treated with antibiotics, and studies to identify alternative products that enhance bacteriological cure are needed. Plant extracts are one of several potential alternative therapeutics [29]. In this exploratory study, we attempted to determine the in vitro inhibitory activity of three common plant extracts. Our results demonstrate that garlic extracts exhibited an in vitro inhibitory effect against *E. coli* and *S. aureus* that had been previously isolated from cases of bovine mastitis in the U.S. These results are like the results of several other studies; however, the MIC and MBC values vary among studies and depend on the origin of garlic, source of isolated bacteria, and the tested bacterial concentrations.

Our results are similar to results of Al Noman et al. (2023) [30], who used thin-layer chromatography with plates coated with thin silicas and reported an MIC for *E. coli* of 0.625 mg/mL [30]. In that study, one *E. coli* isolate, which had been isolated from poultry feces, was revived from stock solution and used for the experiments. Our study is more robust in that we included three replicates of 10 separate *E. coli* isolates.

Other researchers have used a similar method that relies on colorimetric microdilution with different indicators to detect MICs, and have reported greater MIC and MBC values for garlic extract [31,32]. It is difficult to compare studies, however, as the methods, source of isolates, and the origin of the plants varies among studies. In one study, the MIC of garlic extracts for the ATCC isolates *E. coli* and *S. aureus* were both 6.25 mg/mL, but the MBC varied [31]. In that study, the MBC of *E. coli* ranged from 6.25 to 12.5 mg/mL, while the MBC for *S. aureus* ranged from 25 to 50 mg/mL, but it is hard to compare these results to our study as some of their isolates were selected based on known multidrug resistance [31]. The reasons for the one-dilution difference that we observed in the MIC between *E. coli* and *S. aureus* are not known, but could be attributable to differences in the cell wall characteristics of Gram-positive versus Gram-negative bacteria, or due to resistance mechanisms such as efflux pumps. For example, in *E. coli*, allicin is thought to mainly disrupt the glutathione-dependent redox balance and metabolism. In contrast, in *S. aureus*, it preferentially disables thioredoxin systems and cell wall synthesis enzymes, making them more susceptible. Future mechanistic studies are needed to explore the impact of these differences on the inhibitory effects. Differing inhibitory effects have been noted based on the origin of various isolates. Minh et al. (2024) [32] reported that the MIC and MBC values of fecal *E. coli* recovered from chickens, dogs, pheasants, and pigs varied among the species. In that study, they determined the MIC and MBC of garlic extracts using three different varieties grown in Vietnam (Co Don, Hai Duong, Ly Son) for ESBL-producing *E. coli* isolates. The MIC values ranged from 2.35 to 18.75 mg/mL, while the MBC values ranged from 9.38 to 150 mg/mL. The authors concluded that their results varied based on the source of garlic, with each of the different garlic types grown in different regions of Vietnam [32]. Other researchers have shown that the MIC varies depending on virulence of the organism, and documented that the values were much greater for Shiga toxin-producing *E. coli* (ranging from 30 to 140 µL/mL) as compared to less virulent isolates [33].

In our study, we used fresh garlic extract to determine the inhibitory effect against common mastitis pathogens. Previously, garlic extracts were created using different solvents, which may have influenced the MIC values [34]. Akullo et al. (2022) [34] showed that the MIC against *E. coli* and *S. aureus* of garlic extracted using water and ethanol was 2.5 mg/mL, while MIC of methanol-extracted garlic was 10 mg/mL. However, others have reported MIC values of aqueous and ethanolic garlic extracts against *E. coli* that ranged between 0.064 and 0.128 µg/mL [35]. These varying results demonstrate the difficulty in separating methodological differences from true pharmacological effects.

MIC and MBC of various extracts against bacteria are also influenced by the microbial concentration that is tested. In our study, the bacterial concentration that was titrated at 0.5 McFarland was equivalent to 1.5 × 10^8^ CFU/mL. In a separate study, MIC and MBC of fresh garlic extract against *E. coli* titrated at 1 × 10^6^ CFU/mL were both 3.6 mg/mL [36], which were smaller than in our study. However, neither study measured concentration of active metabolites. According to the CLSI guidelines [23], the bacterial concentration for the micro-dilution method test should be 1–2 × 10^8^ CFU/mL to achieve reliable results.

We did not detect any inhibitory effects of fresh ginger or fresh onion extracts against growth of *E. coli* or *S. aureus*. In agreement with our results, concentrations of under 50 mg/mL of onion extracts were shown to lack sterile zones when tested against *E. coli* and *S. aureus*, which had been recovered from wounds of human patients in Nigeria [37]. Those researchers reported that ethanolic and aqueous onion extracts at 50 mg/mL did not produce any sterile zones, and estimated that the MIC was 150 mg/mL for onion extracted using ethanol, while they could not determine the MIC of aqueous extracts of onion against either organism [37]. Using thin-layer chromatography, Al Noman et al. (2023) reported that the MIC of ginger extracts against *E. coli* isolated from poultry was greater than or equal to 9.0 mg/mL [30].

Our study was an exploratory project designed to generate proof of concept, and both methodological and biological effects may have impacted our results. While we used a robust method (broth microdilution) to assess the possible inhibitory effects, there are several potential reasons that our study may not have been able to detect inhibitory properties of the ginger or onion extracts. Extraction issues are sometimes noted as influencing inhibitory properties, but it is unlikely that our extraction method or degradation before testing influenced our results, as we used pure extracts that were tested immediately after extraction. However, we did not attempt to measure any of the active metabolites in the plant extracts, and the concentrations of the active compounds may have been influenced by the time since harvest, storage conditions, or differences among the cultivars. It is also possible that defense mechanisms of the bacteria reduced the inhibitory effects of the plant extracts. The outer membrane of *E. coli* has poor permeability to large compounds, and the use of a solvent may have enhanced their activity. Many bacteria also have efflux systems and the ability to neutralize electrophilic compounds that may have reduced the inhibitory effects. Future studies of these extracts should be designed to address these issues.

Our study has several limitations. This study was designed as an exploratory study, and we did not have access to the strains, cultivars, storage conditions, or country of origin of the plants that we used. The plants were purchased at a market that sources both domestic and imported produce. Most ginger and garlic are imported into the U.S., although many onions and some garlic are grown domestically. Future studies exploring inhibitory properties of plant extracts should be designed to compare various cultivars, especially for garlic, as we observed inhibitory properties of that extract. Another important limitation of our exploratory study is that in vitro studies do not reflect mammary gland conditions. Future studies should determine efficacy of garlic extracts in milk and include in vivo experiments in cows with naturally occurring mastitis.

## 5. Conclusions

Fresh extract of garlic was shown to inhibit *E. coli* and *S. aureus* that had been isolated from milk samples of cows with mastitis. In contrast, extracts of fresh ginger or onion did not inhibit growth of the same organisms. Future studies of botanical compounds should focus on evaluating antimicrobial properties of garlic against additional mastitis pathogens and include studies to determine the ability to achieve inhibitory concentrations in the mammary gland.

## Figures and Tables

**Table 1:** Percent of *E. coli* (n = 10) and *S. aureus* (n = 10) inhibited by garlic at each concentration (bold indicates MIC). Three replicates of each isolate were tested.

Isolates	Dilution Concentration (µL/mL)										Amp ^a^	Cef ^b^
	50	25	12.5	6.25	3.12	1.56	0.8	0.4	0.2	0.1		
*E. coli*												
E1	100	100	100	100	100	**100**	0	0	0	0	100	100
E2	100	100	100	100	100	**100**	0	0	0	0	100	100
E3	100	100	100	100	100	**100**	0	0	0	0	100	100
E4	100	100	100	100	100	**100**	0	0	0	0	100	100
E5	100	100	100	100	100	**100**	0	0	0	0	100	100
E6	100	100	100	100	100	**100**	0	0	0	0	100	100
E7	100	100	100	100	100	**100**	0	0	0	0	100	100
E8	100	100	100	100	100	**100**	0	0	0	0	100	100
E9	100	100	100	100	100	**100**	0	0	0	0	100	100
E10	100	100	100	100	100	**100**	0	0	0	0	100	100
*S. aureus*												
S1	100	100	100	100	**100**	0	0	0	0	0	100	100
S2	100	100	100	100	**100**	0	0	0	0	0	100	100
S3	100	100	100	100	**100**	0	0	0	0	0	100	100
S4	100	100	100	100	100	**100**	0	0	0	0	100	100
S5	100	100	100	100	**100**	0	0	0	0	0	100	100
S6	100	100	100	100	100	**100**	0	0	0	0	100	100
S7	100	100	100	100	**100**	0	0	0	0	0	100	100
S8	100	100	100	100	**100**	0	0	0	0	0	100	100
S9	100	100	100	100	100	**100**	0	0	0	0	100	100
S10	100	100	100	100	100	**100**	0	0	0	0	100	100
E_ATCC_^c^	100	100	100	100	100	**100**	0	0	0	0	100	100
S_ATCC_^d^	100	100	100	100	100	**100**	0	0	0	0	100	100

^a^ ampicillin; ^b^ ceftiofur; ^c^ *E. coli* ATCC 25922; ^d^ *S. aureus* ATCC 25923.

## Data Availability

The original contributions presented in this study are included in the article/Supplementary Material. Further inquiries can be directed to the corresponding author.

## Supplementary Materials

The following supporting information can be downloaded at: https://www.mdpi.com/article/10.3390/vetsci12100947/s1, Table S1: Plate design for herbal plants against *E. coli* and *S. aureus*; Figure S1: Antibacterial activity of *E. coli* and *S. aureus* isolates: A—MIC values and B—MBC values; Figure S2: MIC- and MBC-transformed log2 of *E. coli* and *S. aureus* exposed to garlic extracts: A—log2-transformed MIC and B—log2-transformed MBC.

## Abbreviations

The following abbreviations are used in this manuscript:

MIC	Minimum Inhibitory Concentration
MBC	Minimum Bactericidal Concentration
