# Supplementary material for: Evaluation of In Vitro Inhibitory Activity of Extracts of Garlic, Ginger, and Onion Against Escherichia coli and Staphylococcus aureus Isolated from Milk of Dairy Cows
Source: Vet Sci. 2025 Sep 30;12(10):947. doi: 10.3390/vetsci12100947 (PMC12567945; doi:10.3390/vetsci12100947)
Supplement: Supplementary file 1 [file vetsci-12-00947-s001.zip › vetsci-3846542-supplementary.pdf]

Supplementary Material

Table S1. Plate design for herbal plants against *E. coli* and *S. aureus*.

	1	2	3	4	5	6	7	8	9	10	11	12
A	Ex. 2^{0}, Iso., Re., E1/S1	Ex. 2^{1}, Iso., Re., E1/S1	Ex. 2^{2}, Iso., Re., E1/S1	Ex. 2^{3}, Iso., Re., E1/S1	Ex. 2^{4}, Iso., Re., E1/S1	Ex. 2^{5}, Iso., Re., E1/S1	Ex. 2^{6}, Iso., Re., E1/S1	Ex. 2^{7}, Iso., Re., E1/S1	Ex. 2^{8}, Iso., Re., E1/S1	Ex. 2^{9}, Iso., Re., E1/S1	Am., Iso., Re., E1/S1 (+)	Cef., Iso., Re., E1/S1 (+)
B	Ex. 2^{0}, Iso., Re., E1/S1	Ex. 2^{1}, Iso., Re., E1/S1	Ex. 2^{2}, Iso., Re., E1/S1	Ex. 2^{3}, Iso., Re., E1/S1	Ex. 2^{4}, Iso., Re., E1/S1	Ex. 2^{5}, Iso., Re., E1/S1	Ex. 2^{6}, Iso., Re., E1/S1	Ex. 2^{7}, Iso., Re., E1/S1	Ex. 2^{8}, Iso., Re., E1/S1	Ex. 2^{9}, Iso., Re., E1/S1	Am., Iso., Re., E1/S1 (+)	Cef., Iso., Re., E1/S1 (+)
C	Ex. 2^{0}, Iso., Re., E1/S1	Ex. 2^{1}, Iso., Re., E1/S1	Ex. 2^{2}, Iso., Re., E1/S1	Ex. 2^{3}, Iso., Re., E1/S1	Ex. 2^{4}, Iso., Re., E1/S1	Ex. 2^{5}, Iso., Re., E1/S1	Ex. 2^{6}, Iso., Re., E1/S1	Ex. 2^{7}, Iso., Re., E1/S1	Ex. 2^{8}, Iso., Re., E1/S1	Ex. 2^{9}, Iso., Re., E1/S1	Am., Iso., Re., E2/S2 (+)	Cef., Iso., Re., E2/S2 (+)
D	Ex. 2^{0}, Iso., Re., E2/S2	Ex. 2^{1}, Iso., Re., E2/S2	Ex. 2^{2}, Iso., Re., E2/S2	Ex. 2^{3}, Iso., Re., E2/S2	Ex. 2^{4}, Iso., Re., E2/S2	Ex. 2^{5}, Iso., Re., E2/S2	Ex. 2^{6}, Iso., Re., E2/S2	Ex. 2^{7}, Iso., Re., E2/S2	Ex. 2^{8}, Iso., Re., E2/S2	Ex. 2^{9}, Iso., Re., E2/S2	Am., Iso., Re., E2/S2 (+)	Cef., Iso., Re., E2/S2 (+)
E	Ex. 2^{0}, Iso., Re., E2/S2	Ex. 2^{1}, Iso., Re., E2/S2	Ex. 2^{2}, Iso., Re., E2/S2	Ex. 2^{3}, Iso., Re., E2/S2	Ex. 2^{4}, Iso., Re., E2/S2	Ex. 2^{5}, Iso., Re., E2/S2	Ex. 2^{6}, Iso., Re., E2/S2	Ex. 2^{7}, Iso., Re., E2/S2	Ex. 2^{8}, Iso., Re., E2/S2	Ex. 2^{9}, Iso., Re., E2/S2	LB., Iso., Re., E1/S1 (-)	LB., Iso., Re., E1/S1 (-)
F	Ex. 2^{0}, Iso., Re., E2/S2	Ex. 2^{1}, Iso., Re., E2/S2	Ex. 2^{2}, Iso., Re., E2/S2	Ex. 2^{3}, Iso., Re., E2/S2	Ex. 2^{4}, Iso., Re., E2/S2	Ex. 2^{5}, Iso., Re., E2/S2	Ex. 2^{6}, Iso., Re., E2/S2	Ex. 2^{7}, Iso., Re., E2/S2	Ex. 2^{8}, Iso., Re., E2/S2	Ex. 2^{9}, Iso., Re., E2/S2	LB., Iso., Re., E2/S2 (-)	LB., Iso., Re., E2/S2 (-)
G	Ex. 2^{0}, Iso., Re., E. ATCC	Ex. 2^{1}, Iso., Re., E. ATCC	Ex. 2^{2}, Iso., Re., E. ATCC	Ex. 2^{3}, Iso., Re., E. ATCC	Ex. 2^{4}, Iso., Re., E. ATCC	Ex. 2^{5}, Iso., Re., E. ATCC	Ex. 2^{6}, Iso., Re., E. ATCC	Ex. 2^{7}, Iso., Re., E. ATCC	Ex. 2^{8}, Iso., Re., E. ATCC	Ex. 2^{9}, Iso., Re., E. ATCC	Am., Iso., Re., E. ATCC (+)	Cef., Iso., Re., E. ATCC (+)
H	Ex. 2^{0}, Iso., Re., S. ATCC	Ex. 2^{1}, Iso., Re., S. ATCC	Ex. 2^{2}, Iso., Re., S. ATCC	Ex. 2^{3}, Iso., Re., S. ATCC	Ex. 2^{4}, Iso., Re., S. ATCC	Ex. 2^{5}, Iso., Re., S. ATCC	Ex. 2^{6}, Iso., Re., S. ATCC	Ex. 2^{7}, Iso., Re., S. ATCC	Ex. 2^{8}, Iso., Re., S. ATCC	Ex. 2^{9}, Iso., Re., S. ATCC	Am., Iso., Re., S. ATCC (+)	Cef., Iso., Re., S. ATCC (+)

[A, B, C/D, E, F]Three replicates of each *E. coli* or *S. aureus* tested against each extract. [G,H]ATCC *E. coli* or *S. aureus* tested against each extract. [1-10]Diluted series from 2^0 to 2^9 of each extract against each *E. coli* or *S. aureus* isolate. [11, 12]Positive and negative controls. Ex. = extract, Iso. = iso-sensitive broth, Re. = resazurin, E. = *E. coli*, S. = *S. aureus*, E. ATCC = *E. coli* ATCC 25922, S. ATCC = *S. aureus* ATCC 5923, Am. = ampicillin, Cef. = ceftiofur, (+) = positive controls with bacterial growth, (-) = negative controls without bacterial growth.

A

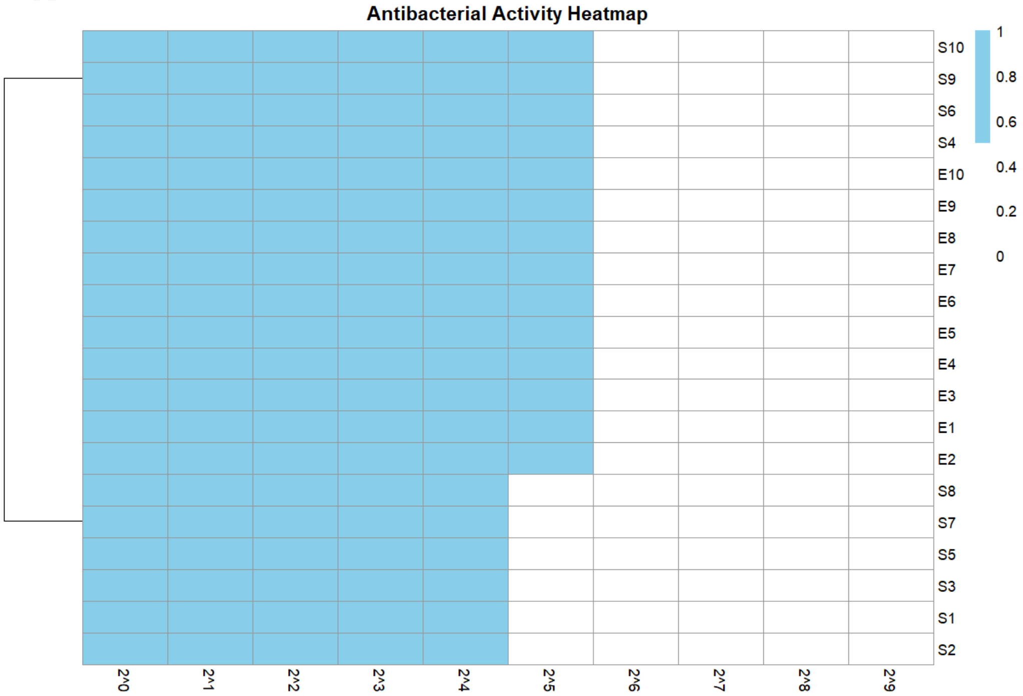

B

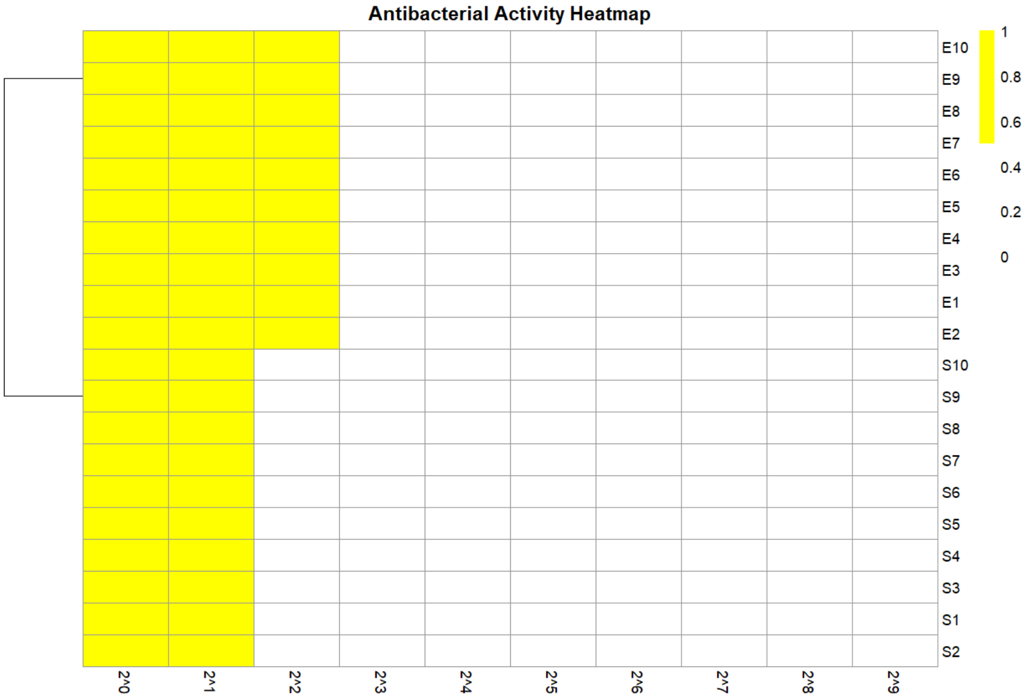

Figure S1. Antibacterial Activity of *E. coli* and *S. aureus* isolates; A – MIC values; B – MBC values.

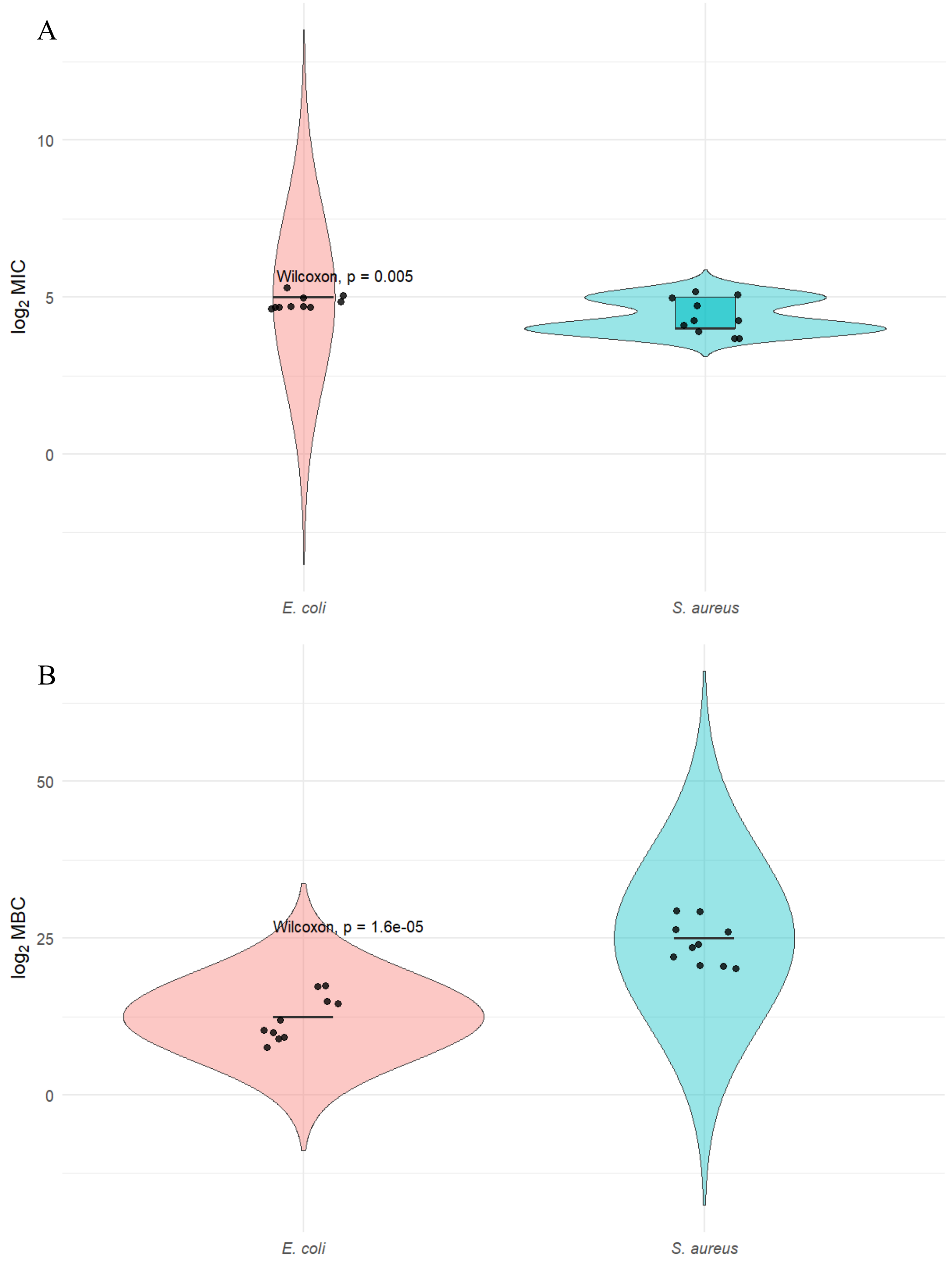

Figure S2. MIC, MBC transformed log_2 of *E. coli* and *S. aureus* exposed to garlic extracts; A – log_2-transformed MIC; B – log_2-transformed MBC.
